# Supplementary material for: Elevated glucose increases genomic instability by inhibiting nucleotide excision repair
Source: Life Sci Alliance. 2021 Aug 23;4(10):e202101159. doi: 10.26508/lsa.202101159 (PMC8385305; doi:10.26508/lsa.202101159)
Supplement: Supplementary file 1 [file LSA-2021-01159_TableS1.docx]

**Table S1.** Primers for qRT-PCR analyses.

| ***Gene* (Protein)** | **mRNA Variants Amplified** | **Forward / Reverse Primer** |
| --- | --- | --- |
| *XPA* | NM_000380.3 | gacacaggaggaggcttcatt / tcgcatattacataatcaaattccata |
| *ERCC3* (XPB) | NM_001303418.1, NM_000122.1, NM_001303416.1 | ttgggcctaagctctacgaa / cactggactttggcgatgta |
| *XPC* | NM_004628.4 | agaaaatgtgcagcgatggt / agaacacctctagccactggtc |
| *ERCC2* (XPD) | NM_000400.3, NM_001130867.1 | catgccaatgtggtggttta / ggacaccaggtctgcaatc |
| *DDB2* (XPE) | NM_000107.2 | catgctgtgattggttcctg / gcaggtcccaaattttcact |
| *ERCC4* (XPF) | NM_005236.2 | tcctgaccagttctctctaccac / tccatgaaatttccttagagcag |
| *ERCC5* (XPG) | NM_000123.3 | ggaagattcgctccatgaat / tgctaagaggttgctctccag |
| *ERCC8* (CSA) | NM_001007233.2, NM_001290285.1, NM_001007234.2, NM_000082.3 | acacatgtaaagcagtgtgttcc / cacactgtatctgtgaacatcagg |
| *ERCC6* (CSB) | NM_001346440.1; NM_000124.3; NM_001277059.1; NM_001277058.1 | gccagatgacaccttttggt / gcttcattaagcatgatttttctg |
| *HIF1A* (HIF-1α) | NM_001243084.1, NM_181054.2, NM_001530.3 | gatagcaagactttcctcagtcg / tcggtaattctttcatcacaataaga |
| *VEGFA* | NM_001171628.1, NM_001033756.2, NM_001171630.1, NM_003376.5, NM_001025366.2, NM_001171625.1, NM_001287044.1, NM_001171626.1, NM_001204384.1, NM_001171627.1 | ccaggaaagactgatacagaacg / tcaggtttctggattaaggactg |
| *PDGFA* | NM_002607.5 | aagcagccaaccagatgtga / ggaggagaacaaagaccgca |
| *HIF1AN* (FIH) | NM_017902.2 | gataccaatatcccctttgcag / ggacatgttccacgtcatgt |
| *IDH1* | NM_005896.3; NM_001282386.1; NM_001282387.1 | ggtgacatacctggtacataactttg / gtgtgcaaaatcttcaattgactt |
| *TUBB* | NM_001293212.1, NM_001293214.1, NM_001293213.1, NM_178014.3, NM_001293215.1, NM_001293216.1 | ttcaatctccctccaagctc / gggaaggattccacttgaca |
